# Correction: Exploring the reproducibility of functional connectivity alterations in Parkinson's disease

**DOI:** 10.1371/journal.pone.0197121

**Published:** 2018-05-03

**Authors:** Liviu Badea, Mihaela Onu, Tao Wu, Adina Roceanu, Ovidiu Bajenaru

In the Methods section, there is an error in the equation in the “Reproducibility of the individual differentiating FC changes” section. Please view the correct equation here:

$$p(\min\left(\max\left(p_{+}\right)\right))=\left|\left\{permutation\;\sigma |min{(\max\left(p_{+}\right))}^{\left(\sigma \right)}\leq \min(max(p_{+}))\right\}\right|/N$$

## References

[1] Badea L, Onu M, Wu T, Roceanu A, Bajenaru O (2017) Exploring the reproducibility of functional connectivity alterations in Parkinson’s disease. PLoS ONE 12(11): e0188196 https://doi.org/10.1371/journal.pone.0188196 29182621 10.1371/journal.pone.0188196 PMC5705108

