# Supplementary material for: Exploring the reproducibility of functional connectivity alterations in Parkinson’s disease
Source: PLoS One. 2017 Nov 28;12(11):e0188196. doi: 10.1371/journal.pone.0188196 (PMC5705108; doi:10.1371/journal.pone.0188196)
Supplement: S1 File — (DOC) [file pone.0188196.s001.doc]

**Supporting Information**

## Resting-state fMRI studies of Parkinson’s disease

**Table A.** Resting-state fMRI studies of Parkinson’s disease

| Study | Main study characteristics | eyes open / closed | NC | PD | Patient characteristics |
| --- | --- | --- | --- | --- | --- |
| Gottlich 2013 | graphs, AAL, AAL subdivision in 343 cortical & subcortical ROIs | eyes closed | 20 | 37 | advanced disease, on medication (L-dopa, agonists) |
| Long 2012 | **classifier:** SVM (ALFF, ReHo, RFCS, GM, WM, CSF) accuracy 86.96%, sensitivity 78.95%, specificity 92.59%, precision 88% | eyes closed | 27 | 19 | early stage (H&Y 1-2), OFF (12 h) |
| Skidmore 2013 | **classifier** (ALFF): accuracy 88%, sensitivity 92%, specificity 87%, precision 87% | eyes closed | 15 | 14 | OFF(12-18 h) |
| Baudrexel 2011 | FC STN, M1 hand area | eyes closed | 44 | 31 | early stage PD patients (n=31) during the medication-off state with healthy controls (n=44); 16 tremor, 15 non-tremor |
| Wu 2011 | FC pre-SMA - M1 seeds: pre-SMA, M1, PCC | eyes closed | 18 | 18 | akinesia right (at most mild tremor), OFF (12h) H&Y 1.78+-0.5 |
| Kwak 2010 | 6 striatal seeds: 3 caudate (inferior ventral striatum, superior ventral striatum, dorsal caudate), 3 putamen (dorsal caudal putamen, dorsal rostral putamen, ventral rostral putamen) | eyes fixed on cross | 24 | 25 | mild to moderate stage (H&Y 1-2.5), ON and OFF(12–18 h) |
| Kwak 2012 | ALFF (fALLF) |  | 24 | 24 | mild to moderate stage (H&Y 1-2.5) ON and OFF (12-18h) |
| Helmich 2010 | FC anterior, posterior putamen, caudate posterior cingulate (control) | eyes closed | 36 | 41 | 13 PD without any tremor, 18 moderate to severe. 10 - never anti-PD medication (median H&Y 2.1, max 5) perfectly age-matched (57 years) |
| Helmich 2011 | tremor | eyes closed | 36 | 41 | 19 tremor, 23 nontremor, right-handed, 12 no medication, OFF (12 h) |
| Luo 2014 | FC anterior, posterior putamen, anterior caudate, amygdala | eyes closed | 52 | 52 | 52 PD right handed, early stage drug-naïve, H&Y 1.85 (max 3), 31 right onset, 21 left onset |
| Yu 2013 | FC putamen, caudate, and SMA |  | 20 | 19 | OFF obvious at least a mild tremor |
| Hacker 2012 | FC striatum 6 ROIs: (i) caudate nucleus; (ii) anterior putamen; and (iii) posterior putamen | eyes open (fixate cross) | 19 | 13 | advanced PD **ON** |
| Kurani 2015 | FC (restricted to motor areas) seeds: STN (most affected part), posterior cingulate (control seed) | eyes open (focused on the word “RELAX”) | 19 | 39 | 20 de novo 19 moderate OFF |
| Baggio 2015 | cognitive impairement ICA+dual regression (25 networks), group comparison of: DMN, dorsal attention network (DAN), bilateral frontoparietal networks (FPN), 43 seeds (10 DAN, 18 DMN, 15 FPN) |  | 36 | 65 | ON state 65 nondemented: 34% mild cognitive impairement (MCI) |
| Esposito 2013 | ICA (fastICA BrainVoyager 40 components, restriction to sensorimotor network-best fit with previous template) | eyes closed | 18 | 20 | 20 drug naïve PD, 10 before & after (1H) levodopa, 10 before & after placebo |
| Szewczyk-Krolikowski 2014 | ICA+dual regression – basal ganglia (BG) network from 80 separate controls - classifier based on average BG component values in voxels discriminating PD(OFF)-controls | eyes open | 19 | 19 | discovery cohort: 19 PD ON/OFF, 19 controls validation cohort: 13 PD (5 drug-naïve), no controls (this disallows evaluation of specificity!) 80 elderly controls for BG template (MELODIC groupICA 50 components) all subjects right-handed |
| Tessitore 2012 | fastICA, sogICA  40 components select DMN, the frontoparietal (right and left FPN), sensorimotor network (SMN), visual, auditory | eyes closed | 15 | 29 | PD ON 16 FOG+ |
| Sharman 2013 | ROIs: caudate, putamen, globus pallidus, thalamus sensorimotor (M1, postcentral gyrus) associative (ventrolateral, dorsolateral prefrontal) limbic (orbitofrontal, rectus gyrus, cingulate, insula, medial temporal ctx, perirhinal & entorhinal cortex, hippocampus, amygdala) | eyes closed | 45 | 36 |  |
| Wu 2012 | ROIs: SNc bilaterally  effective connectivity Granger causality analysis (GCA) with long TR=2000 & short TR=400 | eyes closed | 16 | 16 | 16 de novo OFF/ON |
| Liu 2013 | FC dentate nucleus (cerebellum) -> cerebellar output | eyes closed | 18 | 18 | mild to moderate (1.34 H&Y average) in OFF state 8 rigidity & bradykinesia-dominant (PD_AR)  10 tremor-dominant (PD_T) |
| Chen 2015 | SVM classifier LOOCV based on FC between 116 ROIs of AAL parcellation (150 features selected by Kendall tau correlation) - 93.62% accuracy, 90.47% sensitivity, 96.15 specificity | eyes closed | 26 | 21 | 21 PD (10 males, 11 females, 58.3 years), 26 HC (10 males, 16 females, 61.3 years) OFF state (12 hours) UPDRS 29.8 (sd 9.1), disease duration 3.2 years (sd 3.2) |
| Wen 2013 | depression ALFF | eyes closed | 21 | 33 | OFF 17 depression |

## Datasets

### NEUROCON

The NEUROCON study enrolled 27 patients with Parkinson’s disease (mean age±SD 68.7±10.6 years) and 16 age-matched normal controls (67.6±11.9 years) with no history of neurological or psychiatric disease. The patients were clinically assessed at the Neurology Department of the University Emergency Hospital Bucharest (Romania) to be in the early or moderate stage of the disease according to the Queen Square Brain Bank (QSBB) clinical criteria and met the EFNS/MDS-ES (European Federation of Neurological Societies/Movement Disorder Society–European Section) recommendations for diagnosis of Parkinson’s disease. The mean disease duration was 4.6 (±6.5) years for the entire patient cohort and respectively 2.75 (±2.15) years after excluding three patients with particularly long disease durations (over 10 years: 11, 16 and 32 years, respectively). Despite the longer disease durations, the above-mentioned 3 patients met the criteria for moderately advanced disease (H&Y stage 2) and thus were included in the study. The mean Hoehn and Yahr (H&Y) score [Hoehn and Yahr, 2001] was 1.93 (±0.33) and respectively 1.92 (±0.35) after excluding the 3 patients with long disease durations. All patients were in an early to moderate stage of disease (stages 1 to 2.5). The mean score on the motor subset of the Unified Parkinson’s Disease Rating Scale (UPDRS) [Fahn, 1986] in the off medication condition was 28.3 (±9.3) for the entire patient cohort and 26.9 (±8.8) after excluding the 3 patients with long disease durations. The study has been approved by the University Emergency Hospital Bucharest ethics committee in accordance with the ethical standards of the 1964 Declaration of Helsinki and its later amendments. All patients gave their written informed consent to participate in the study.

**Scanning.** All subjects underwent two consecutive 8 min fMRI scans in a 1.5-Tesla Siemens Avanto MRI scanner, in an awake resting state with their eyes closed. Two consecutive replicate scans were acquired for each subject to enable the study of the reproducibility and respectively homogeneity of FC changes in PD. (A single (control) subject could only be scanned once.) The patients were scanned in the “off medication” state, at least 10 hours after the last intake of their medication. The scanning protocol involved an Echo Planar sequence with repetition time (TR) 3480 ms, echo time (TE) 50 ms, axial orientation, voxel size 3.8×3.8×5 mm (without slice gaps), flip angle 90° and number of averages=1. Each resting state session lasted 8.05 min, comprising 137 volumes. To enable better co-registration to the standard MNI template, high-resolution T1-weighted images were also obtained for all subjects using an MPRAGE sequence (IR method, TR=1940ms, TE=3.08ms, inversion time (IT)=1100ms, voxel size 0.97×0.97×1 mm, number of averages=1).

### Tao Wu

The dataset comprised 20 PD patients (11 males, mean age±SD 65.2±4.4 years) and 20 age-matched normal controls (12 males, 64.8±5.6 years). All patients were in the early to moderate stage of the disease (Hoehn and Yahr stages 1 to 2.5, except for a single patient with H&Y stage 3) and had normal Mini-Mental State Examination (MMSE) scores. Moreover, there was no statistically significant MMSE difference (p=0.43) between PD patients (28.8±1.1) and normal controls (29.1±1.3).

**Scanning.** Both resting state fMRI and anatomic T1 scans were acquired for the 40 subjects in a Siemens Magnetom Trio 3T equipment, in an awake resting state with their eyes closed. Each resting state session lasted about 8 min (239 volumes, TR=2s, TE=40 ms, flip angle=90°) with a voxel size of 4×4×5 mm (64×64 matrix, 28 slices, field of view=256mm×256mm). MPRAGE scans were also obtained (voxel size 1×1×1 mm) for registration to the MNI template.

### PPMI

Imaging data for 91 PD patients (63 males) and 18 normal controls (14 males) were downloaded from the Parkinson’s Progression Markers Initiative (PPMI) study data portal (http://www.ppmi-info.org/access-data-specimens/download-data/, https://ida.loni.usc.edu/home/projectPage.jsp?project=PPMI) with the kind permission of the PPMI consortium. The study includes patients with a diagnosis of PD for two years or less and who are not taking PD medications. The patients and controls are age-matched (p=0.17, mean age±SD 61.3±10.2 years for the PD patients and respectively 64.7±9.7 years for the normal controls). The patients had a mean Hoehn & Yahr score of 1.72 (SD 0.48), with a mean disease duration (at the time of the scan) of 1.9 years (SD 1.0). All patients had H&Y scores 1 to 2, except for only two, who were classified as H&Y stage 3.

**Scanning.** The subjects were scanned in 8 different centers, but with a similar protocol on Siemens Tim Trio 3Tesla scanners. Each resting state session lasted about 8.4 min (210 volumes, TR=2.4s, TE=25ms, flip angle 80°) with a voxel size of 3.3×3.3×3.3 mm (68×66 matrix, 40 slices). Subjects were instructed to rest quietly, keeping their eyes open and not to fall asleep. MPRAGE scans were also obtained (voxel size 1×1×1 mm, TR=2.3s, TE=2.98ms, flip angle=9°) for registration to the MNI template.

Although our functional connectivity computations did not require any particular type of data normalization (as only inter-region correlations are computed, rather than amplitudes), we also considered a subset of scans acquired in a single center (center number 32, with the largest number of PD patient and normal control scans), referred to in the following by the suffix ‘center32’.

## Subject motion in scanner

Since subject motion in the scanner has been observed to have significant influence on the functional connectivities computed from rs-fMRI data, despite motion correction (e.g. [Power et al., 2015]), we also considered subsets of scans with low in-scanner motion (marked by the suffix ‘0’, e.g. ‘NC0’ and ‘PD0’ – see also Table B). The 3 datasets have different in-scanner motion characteristics.

**Table B.** The subsets of scans with low in-scanner motion

| Dataset | Low motion condition | NC scans | PD scans | NC0 scans | PD0 scans |
| --- | --- | --- | --- | --- | --- |
| NEUROCON | max abs motion ≤ 1/4 voxel = 0.958mm  max rel motion ≤ 1/6 voxel = 0.638mm | 31 | 54 | 27 | 39 |
| Tao Wu | max abs motion ≤ 1/4 voxel = 1mm  max rel motion ≤ 1/6 voxel = 0.67mm | 20 | 20 | 13 | 14 |
| PPMI | max abs motion ≤ 1/3 voxel = 1.098mm  max rel motion ≤ 1/3 voxel = 1.098mm  mean abs motion ≤ 1/6 voxel = 0.549mm  max rel motion ≤ 1/6 voxel = 0.549mm | 19 | 134 | 13 | 89 |

The following Tables show the p-values corresponding to potential group differences in motion in the 3 datasets analyzed:

**NEUROCON**

NC-PD (31-54) p-value (t-value)

| p(mean rel) | p(mean abs) | p(max rel) | p(max abs) |
| --- | --- | --- | --- |
| 0.00243021  (-3.14036) | 0.0545481  (-1.96039) | 0.0945346  (-1.6961) | 0.135713  (-1.50937) |

NC0-PD0 (27-39)

| p(mean rel) | p(mean abs) | p(max rel) | p(max abs) |
| --- | --- | --- | --- |
| 0.166962  (-1.39829) | 0.043621  (-2.06138) | 0.319776  (-1.00337) | 0.100148  (-1.67026) |

Note that in the NEUROCON cohort, patients move more than normal controls.

**Tao Wu**

NC-PD (20-20)

| p(mean rel) | p(mean abs) | p(max rel) | p(max abs) |
| --- | --- | --- | --- |
| 0.0270361  (2.33514) | 0.727106  (0.352521) | 0.0074933  (2.96994) | 0.543508  (0.613522) |

NC0-PD0 (13-14)

| p(mean rel) | p(mean abs) | p(max rel) | p(max abs) |
| --- | --- | --- | --- |
| 0.256895  (1.16397) | 0.497445  (0.688602) | 0.0859714  (1.80121) | 0.455533  (0.758043) |

In the Tao Wu cohort, patients move less than normal controls.

**PPMI**

NC-PD (19-134)

| p(mean rel) | p(mean abs) | p(max rel) | p(max abs) |
| --- | --- | --- | --- |
| 0.0619124  (1.9849) | 0.485967  (0.710965) | 0.107866  (1.69169) | 0.134632  (1.56558) |

NC0-PD0 (13-89)

| p(mean rel) | p(mean abs) | p(max rel) | p(max abs) |
| --- | --- | --- | --- |
| 0.0656489 (1.94269) | 0.314195  (-1.03452) | 0.224242  (1.26499) | 0.53616  (0.63367) |

NC_center32-PD_center32 (9-30)

| p(mean rel) | p(mean abs) | p(max rel) | p(max abs) |
| --- | --- | --- | --- |
| 0.242348  (1.23728) | 0.77672  (0.289486) | 0.344483  (1.00257) | 0.318376  (1.05993) |

NC0_center32-PD0_center32 (7-23)

| p(mean rel) | p(mean abs) | p(max rel) | p(max abs) |
| --- | --- | --- | --- |
| 0.173686  (1.40637) | 0.649951  (0.46271) | 0.252684  (1.21065) | 0.13755  (1.64485) |

## ROI-pairs with significant group differences in the separate datasets

Table C below shows the numbers of significant ROI pairs for several significance thresholds for the unpaired t-test between patient and control functional connectivities (for the AAL parcellation and without correction for multiple comparisons, due to the limited sample sizes). A single scan for each subject was considered in this comparison.

**Table C.** Number of significant ROI-pairs for the AAL parcellation

| significance level | NEUROCON(16-27) | TaoWu(20-20) | PPMI(18-91) |
| --- | --- | --- | --- |
| 0.001 | 4 | 15 | 19 |
| 0.005 | 23 | 68 | 81 |
| 0.01 | 48 | 121 | 156 |
| 0.05 | 283 | 489 | 737 |

As expected, the power of the tests was greater for the datasets with more samples, but the effect sizes (t-values) were similar, as can be seen in Table D below, which shows the top positive and respectively negative effect sizes observed.

**Table D.** Top effect sizes (t-values) and the corresponding p-values for the top positive and respectively negative FC alterations

|  | NEUROCON(16-27) | TaoWu(20-20) | PPMI(18-91) |
| --- | --- | --- | --- |
| top t_+_ | 3.05647 | 4.632511 | 4.70764 |
| top p_+_ | 0.00429499 | 4.23E-05 | 7.99E-05 |
| top t_−_ | -4.495779 | -2.91725 | -3.870344 |
| top p_−_ | 6.09E-05 | 0.0062734 | 0.0003765 |

## Comparison of random splits of an ‘eyes open-eyes closed’ dataset

Besides the potentially heterogeneous contrast between PD and normal controls, we also tested our method on a different, potentially more homogeneous contrast, namely ‘eyes open’ versus ‘eyes closed’ resting state in healthy volunteers. We used the *Beijing eyes-open-eyes closed (EO-EC) dataset* [Liu et al., 2013] (http://fcon_1000.projects.nitrc.org/indi/IndiPro.html), which involved 48 college students aged 19–31 years, 24 female with no history of neurological and psychiatric disorders. Each participant underwent three 8 min resting state scanning sessions: an EC session followed by two sessions counter-balanced across subjects: one EO resting state and one EC resting state session.

The functional images were obtained on a Siemens Trio 3 Tesla scanner using an echo-planar imaging sequence with the following parameters: 33 axial slices, thickness/gap=3.5/0.7 mm, in-plane resolution=64×64, repetition time=2000 ms, echo time=30 ms, flip angle=90°, field of view (FOV)=200×200mm^2^, 240 volumes per scan. In addition, a 3D T1-weighted MPRAGE image was acquired with the following parameters: 128 sagittal slices, slice thickness/gap=1.33/0 mm, in-plane resolution=256×192, TR=2530 ms, TE=3.39 ms, inversion time (TI)=1100 ms, flip angle=7°, FOV=256×256 mm^2^. Note that the parameters used in this study are quite similar to the ones from the PD datasets, including the scanning time (~8 min), with the exception of the 1.5 Tesla field strength used in the NEUROCON study (all the other studies used 3 Tesla machines).

We repeated our analyses of reproducibility of group changes in functional connectivity on random splits of the Beijing EO-EC dataset on both “split subjects” (heterogeneous) and “split replicates” (homogeneous) datasets using the AAL parcellation. As in the case of PD, permutation tests were employed to compute p-values of the reproducibility across split datasets. Additionally, we repeated the analysis for the data with global signal regression.

### ‘Eyes Open-Eyes Closed’ FC changes are reproducible

The fact that the well-known *clinical* heterogeneity of Parkinson’s disease is also accompanied by heterogeneity in resting state *functional connectivity* may not *retrospectively* be a big surprise to an experienced neurologist, although its exact extent could not have been estimated a priori, before analyzing the data.

However, does this FC heterogeneity in PD also imply the lack of practical usefulness of rs-fMRI functional connectivity? Are there any other conditions that can be reliably differentiated using resting state functional connectivity? To answer these questions, we applied our approach to a different, potentially more homogeneous contrast, namely that between eyes open and eyes closed resting state conditions in healthy volunteers. Repeating our analysis of reproducibility of FC group changes on random splits of the Beijing eyes open-eyes closed dataset [Liu et al., 2013] revealed *reproducibility* (p<0.05) not just in the *homogeneous* dataset splits, but also in the *heterogeneous* ones (Fig A – only 6% of the heterogeneous and just 0.8% of the homogeneous random splits were non-reproducible at the p>0.05 level). This implies that the EO-EC contrast produces more homogeneous and reproducible global FC changes.


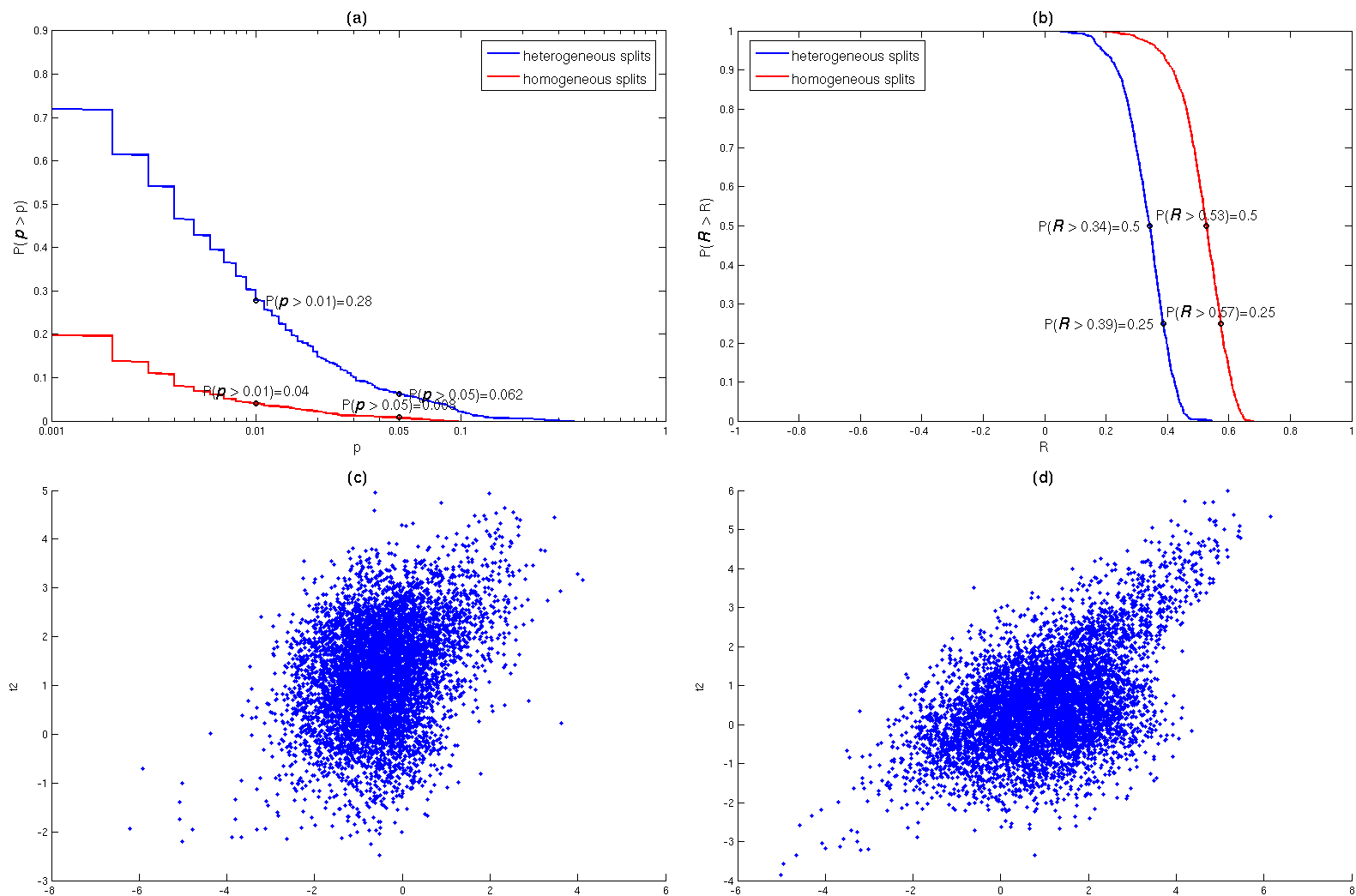


Fig A. *Consistent* reproducibility of ‘eyes open’-‘eyes closed’ (EO-EC) FC changes in random *heterogeneous* and *homogeneous* dataset splits. (A) Complementary cumulative distribution function (CCDF=1-CDF) of the reproducibility p-values for *N_s_*=1056 random *heterogeneous* splits and *N_s_*=814 random *homogeneous* splits. (B) CCDF of the reproducibility measure *R*. (C) A scatter-plot of ROI-pair t-values for a random *heterogeneous* split*.* (D) A scatter-plot of ROI-pair t-values for a random *homogeneous* split*.*

***Learning classifiers for discriminating PD-related FC changes***


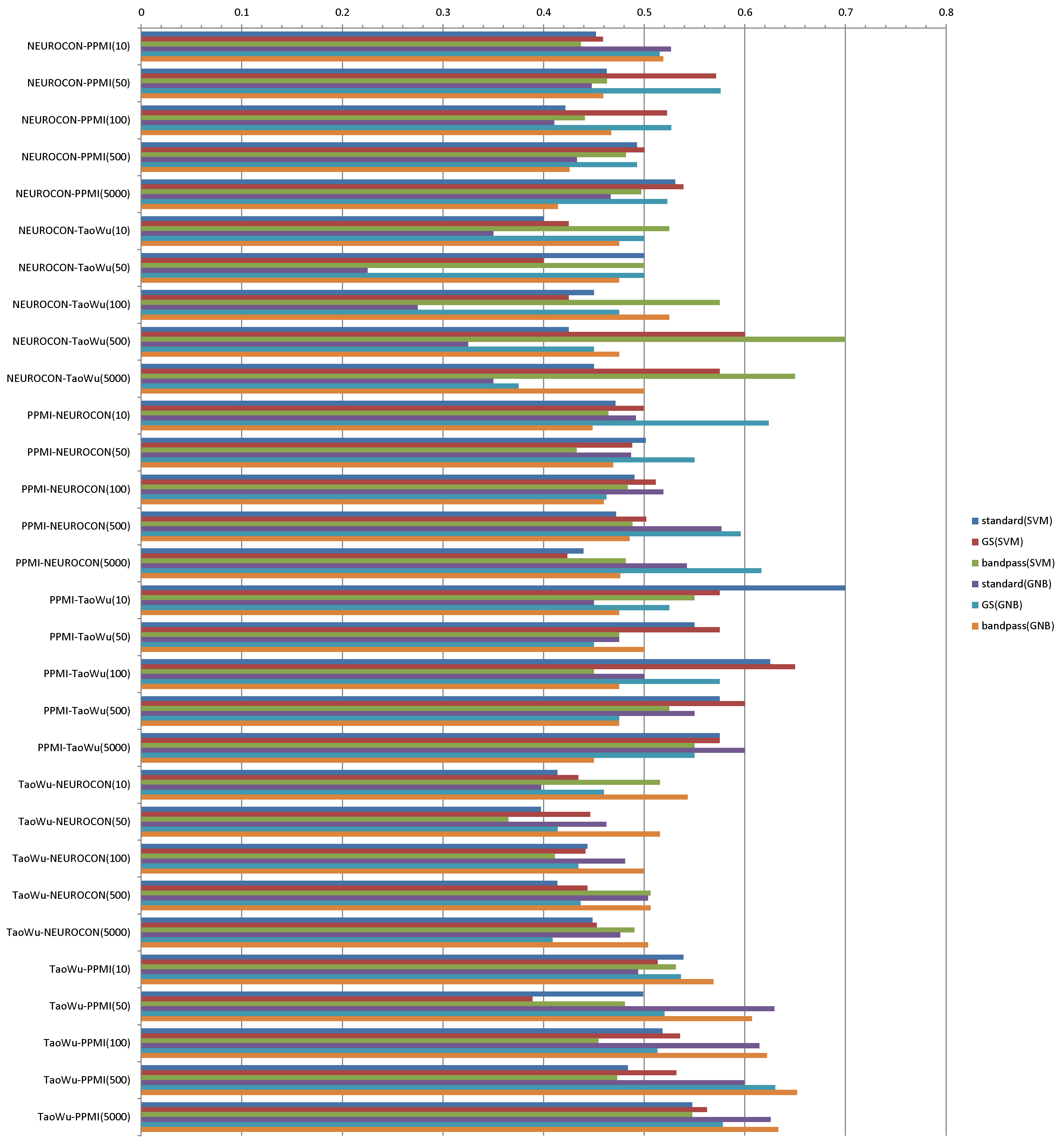


**Fig B. Average accuracies *Aacc* = (*acc*(NC)+acc(PD))/2 for classifiers trained on dataset 1 and tested on dataset 2 for all dataset pairs** using standard preprocessing (‘standard’), global signal regression (GS) and respectively bandpass filtering (0.01-0.1Hz). Classifiers were trained with *N*=10,50,100,500,5000 features. For example, NEUROCON-PPMI(10) shows average accuracies of classifiers trained on NEUROCON and tested on PPMI data using *N*=10 features. SVM – linear SVM classifier, GNB – Gaussian Naïve Bayes classifier.

**Table E**. Detailed performance metrics for the various classifiers

| TRAIN DATASET | TEST DATASET | classifier | N pairs | Pre-processing | TEST AvgaccPerClass | TEST accPerClass(NC) | TEST accPerClass(PD) |
| --- | --- | --- | --- | --- | --- | --- | --- |
| NEUROCON | PPMI | gnb_pooled | 10 | standard | 0.526709 | 0.1579 | 0.895522 |
| NEUROCON | PPMI | svm_linear | 10 | standard | 0.452082 | 0.1579 | 0.746269 |
| NEUROCON | PPMI | gnb_pooled | 50 | standard | 0.447761 | 0 | 0.895522 |
| NEUROCON | PPMI | svm_linear | 50 | standard | 0.462687 | 0 | 0.925373 |
| NEUROCON | PPMI | gnb_pooled | 100 | standard | 0.410644 | 0.05263 | 0.768657 |
| NEUROCON | PPMI | svm_linear | 100 | standard | 0.421642 | 0 | 0.843284 |
| NEUROCON | PPMI | gnb_pooled | 500 | standard | 0.433032 | 0.05263 | 0.813433 |
| NEUROCON | PPMI | svm_linear | 500 | standard | 0.49293 | 0.10526 | 0.880597 |
| NEUROCON | PPMI | gnb_pooled | 5000 | standard | 0.466614 | 0.05263 | 0.880597 |
| NEUROCON | PPMI | svm_linear | 5000 | standard | 0.530833 | 0.26316 | 0.798507 |
| NEUROCON | TaoWu | gnb_pooled | 10 | standard | 0.35 | 0.2 | 0.5 |
| NEUROCON | TaoWu | svm_linear | 10 | standard | 0.4 | 0.15 | 0.65 |
| NEUROCON | TaoWu | gnb_pooled | 50 | standard | 0.225 | 0.15 | 0.3 |
| NEUROCON | TaoWu | svm_linear | 50 | standard | 0.5 | 0.15 | 0.85 |
| NEUROCON | TaoWu | gnb_pooled | 100 | standard | 0.275 | 0.15 | 0.4 |
| NEUROCON | TaoWu | svm_linear | 100 | standard | 0.45 | 0.05 | 0.85 |
| NEUROCON | TaoWu | gnb_pooled | 500 | standard | 0.325 | 0.2 | 0.45 |
| NEUROCON | TaoWu | svm_linear | 500 | standard | 0.425 | 0.05 | 0.8 |
| NEUROCON | TaoWu | gnb_pooled | 5000 | standard | 0.35 | 0.25 | 0.45 |
| NEUROCON | TaoWu | svm_linear | 5000 | standard | 0.45 | 0.1 | 0.8 |
| PPMI | NEUROCON | gnb_pooled | 10 | standard | 0.491637 | 0.6129 | 0.37037 |
| PPMI | NEUROCON | svm_linear | 10 | standard | 0.471326 | 0.3871 | 0.555556 |
| PPMI | NEUROCON | gnb_pooled | 50 | standard | 0.486858 | 0.67742 | 0.296296 |
| PPMI | NEUROCON | svm_linear | 50 | standard | 0.501493 | 0.35484 | 0.648148 |
| PPMI | NEUROCON | gnb_pooled | 100 | standard | 0.519116 | 0.74194 | 0.296296 |
| PPMI | NEUROCON | svm_linear | 100 | standard | 0.490143 | 0.25807 | 0.722222 |
| PPMI | NEUROCON | gnb_pooled | 500 | standard | 0.576762 | 0.83871 | 0.314815 |
| PPMI | NEUROCON | svm_linear | 500 | standard | 0.471924 | 0.12903 | 0.814815 |
| PPMI | NEUROCON | gnb_pooled | 5000 | standard | 0.542413 | 0.67742 | 0.407407 |
| PPMI | NEUROCON | svm_linear | 5000 | standard | 0.439665 | 0.06452 | 0.814815 |
| PPMI | TaoWu | gnb_pooled | 10 | standard | 0.45 | 0.85 | 0.05 |
| PPMI | TaoWu | svm_linear | 10 | standard | 0.7 | 0.6 | 0.8 |
| PPMI | TaoWu | gnb_pooled | 50 | standard | 0.475 | 0.8 | 0.15 |
| PPMI | TaoWu | svm_linear | 50 | standard | 0.55 | 0.3 | 0.8 |
| PPMI | TaoWu | gnb_pooled | 100 | standard | 0.5 | 0.85 | 0.15 |
| PPMI | TaoWu | svm_linear | 100 | standard | 0.625 | 0.35 | 0.9 |
| PPMI | TaoWu | gnb_pooled | 500 | standard | 0.55 | 0.9 | 0.2 |
| PPMI | TaoWu | svm_linear | 500 | standard | 0.575 | 0.15 | 1 |
| PPMI | TaoWu | gnb_pooled | 5000 | standard | 0.6 | 0.85 | 0.35 |
| PPMI | TaoWu | svm_linear | 5000 | standard | 0.575 | 0.15 | 1 |
| TaoWu | NEUROCON | gnb_pooled | 10 | standard | 0.397551 | 0.25807 | 0.537037 |
| TaoWu | NEUROCON | svm_linear | 10 | standard | 0.41368 | 0.29032 | 0.537037 |
| TaoWu | NEUROCON | gnb_pooled | 50 | standard | 0.462366 | 0.25807 | 0.666667 |
| TaoWu | NEUROCON | svm_linear | 50 | standard | 0.397252 | 0.3871 | 0.407407 |
| TaoWu | NEUROCON | gnb_pooled | 100 | standard | 0.480884 | 0.25807 | 0.703704 |
| TaoWu | NEUROCON | svm_linear | 100 | standard | 0.443548 | 0.3871 | 0.5 |
| TaoWu | NEUROCON | gnb_pooled | 500 | standard | 0.503883 | 0.32258 | 0.685185 |
| TaoWu | NEUROCON | svm_linear | 500 | standard | 0.41368 | 0.29032 | 0.537037 |
| TaoWu | NEUROCON | gnb_pooled | 5000 | standard | 0.476105 | 0.32258 | 0.62963 |
| TaoWu | NEUROCON | svm_linear | 5000 | standard | 0.448626 | 0.19355 | 0.703704 |
| TaoWu | PPMI | gnb_pooled | 10 | standard | 0.493912 | 0.36842 | 0.619403 |
| TaoWu | PPMI | svm_linear | 10 | standard | 0.539081 | 0.47368 | 0.604478 |
| TaoWu | PPMI | gnb_pooled | 50 | standard | 0.629419 | 0.68421 | 0.574627 |
| TaoWu | PPMI | svm_linear | 50 | standard | 0.499018 | 0.73684 | 0.261194 |
| TaoWu | PPMI | gnb_pooled | 100 | standard | 0.614493 | 0.68421 | 0.544776 |
| TaoWu | PPMI | svm_linear | 100 | standard | 0.518068 | 0.84211 | 0.19403 |
| TaoWu | PPMI | gnb_pooled | 500 | standard | 0.599568 | 0.68421 | 0.514925 |
| TaoWu | PPMI | svm_linear | 500 | standard | 0.483896 | 0.68421 | 0.283582 |
| TaoWu | PPMI | gnb_pooled | 5000 | standard | 0.625687 | 0.68421 | 0.567164 |
| TaoWu | PPMI | svm_linear | 5000 | standard | 0.547722 | 0.78947 | 0.30597 |
| NEUROCON | PPMI | gnb_pooled | 10 | GS | 0.515318 | 0.10526 | 0.925373 |
| NEUROCON | PPMI | svm_linear | 10 | GS | 0.458955 | 0 | 0.91791 |
| NEUROCON | PPMI | gnb_pooled | 50 | GS | 0.575805 | 0.31579 | 0.835821 |
| NEUROCON | PPMI | svm_linear | 50 | GS | 0.571485 | 0.1579 | 0.985075 |
| NEUROCON | PPMI | gnb_pooled | 100 | GS | 0.526905 | 0.21053 | 0.843284 |
| NEUROCON | PPMI | svm_linear | 100 | GS | 0.522584 | 0.05263 | 0.992537 |
| NEUROCON | PPMI | gnb_pooled | 500 | GS | 0.49293 | 0.10526 | 0.880597 |
| NEUROCON | PPMI | svm_linear | 500 | GS | 0.500393 | 0.10526 | 0.895522 |
| NEUROCON | PPMI | gnb_pooled | 5000 | GS | 0.522977 | 0.1579 | 0.88806 |
| NEUROCON | PPMI | svm_linear | 5000 | GS | 0.538885 | 0.42105 | 0.656716 |
| NEUROCON | TaoWu | gnb_pooled | 10 | GS | 0.5 | 0.2 | 0.8 |
| NEUROCON | TaoWu | svm_linear | 10 | GS | 0.425 | 0.15 | 0.7 |
| NEUROCON | TaoWu | gnb_pooled | 50 | GS | 0.5 | 0.4 | 0.6 |
| NEUROCON | TaoWu | svm_linear | 50 | GS | 0.4 | 0.05 | 0.75 |
| NEUROCON | TaoWu | gnb_pooled | 100 | GS | 0.475 | 0.45 | 0.5 |
| NEUROCON | TaoWu | svm_linear | 100 | GS | 0.425 | 0 | 0.85 |
| NEUROCON | TaoWu | gnb_pooled | 500 | GS | 0.45 | 0.6 | 0.3 |
| NEUROCON | TaoWu | svm_linear | 500 | GS | 0.6 | 0.25 | 0.95 |
| NEUROCON | TaoWu | gnb_pooled | 5000 | GS | 0.375 | 0.55 | 0.2 |
| NEUROCON | TaoWu | svm_linear | 5000 | GS | 0.575 | 0.15 | 1 |
| PPMI | NEUROCON | gnb_pooled | 10 | GS | 0.623656 | 0.58065 | 0.666667 |
| PPMI | NEUROCON | svm_linear | 10 | GS | 0.499403 | 0.25807 | 0.740741 |
| PPMI | NEUROCON | gnb_pooled | 50 | GS | 0.549881 | 0.45161 | 0.648148 |
| PPMI | NEUROCON | svm_linear | 50 | GS | 0.488053 | 0.16129 | 0.814815 |
| PPMI | NEUROCON | gnb_pooled | 100 | GS | 0.462366 | 0.25807 | 0.666667 |
| PPMI | NEUROCON | svm_linear | 100 | GS | 0.51135 | 0.09677 | 0.925926 |
| PPMI | NEUROCON | gnb_pooled | 500 | GS | 0.595878 | 0.58065 | 0.611111 |
| PPMI | NEUROCON | svm_linear | 500 | GS | 0.502091 | 0.09677 | 0.907407 |
| PPMI | NEUROCON | gnb_pooled | 5000 | GS | 0.616487 | 0.67742 | 0.555556 |
| PPMI | NEUROCON | svm_linear | 5000 | GS | 0.423536 | 0.03226 | 0.814815 |
| PPMI | TaoWu | gnb_pooled | 10 | GS | 0.525 | 0.45 | 0.6 |
| PPMI | TaoWu | svm_linear | 10 | GS | 0.575 | 0.4 | 0.75 |
| PPMI | TaoWu | gnb_pooled | 50 | GS | 0.45 | 0.45 | 0.45 |
| PPMI | TaoWu | svm_linear | 50 | GS | 0.575 | 0.3 | 0.85 |
| PPMI | TaoWu | gnb_pooled | 100 | GS | 0.575 | 0.45 | 0.7 |
| PPMI | TaoWu | svm_linear | 100 | GS | 0.65 | 0.35 | 0.95 |
| PPMI | TaoWu | gnb_pooled | 500 | GS | 0.475 | 0.35 | 0.6 |
| PPMI | TaoWu | svm_linear | 500 | GS | 0.6 | 0.2 | 1 |
| PPMI | TaoWu | gnb_pooled | 5000 | GS | 0.55 | 0.55 | 0.55 |
| PPMI | TaoWu | svm_linear | 5000 | GS | 0.575 | 0.2 | 0.95 |
| TaoWu | NEUROCON | gnb_pooled | 10 | GS | 0.459976 | 0.29032 | 0.62963 |
| TaoWu | NEUROCON | svm_linear | 10 | GS | 0.434588 | 0.25807 | 0.611111 |
| TaoWu | NEUROCON | gnb_pooled | 50 | GS | 0.413978 | 0.16129 | 0.666667 |
| TaoWu | NEUROCON | svm_linear | 50 | GS | 0.446237 | 0.22581 | 0.666667 |
| TaoWu | NEUROCON | gnb_pooled | 100 | GS | 0.434588 | 0.25807 | 0.611111 |
| TaoWu | NEUROCON | svm_linear | 100 | GS | 0.441458 | 0.29032 | 0.592593 |
| TaoWu | NEUROCON | gnb_pooled | 500 | GS | 0.436679 | 0.35484 | 0.518519 |
| TaoWu | NEUROCON | svm_linear | 500 | GS | 0.443548 | 0.3871 | 0.5 |
| TaoWu | NEUROCON | gnb_pooled | 5000 | GS | 0.408901 | 0.35484 | 0.462963 |
| TaoWu | NEUROCON | svm_linear | 5000 | GS | 0.452808 | 0.3871 | 0.518519 |
| TaoWu | PPMI | gnb_pooled | 10 | GS | 0.536332 | 0.73684 | 0.335821 |
| TaoWu | PPMI | svm_linear | 10 | GS | 0.513354 | 0.57895 | 0.447761 |
| TaoWu | PPMI | gnb_pooled | 50 | GS | 0.520228 | 0.42105 | 0.619403 |
| TaoWu | PPMI | svm_linear | 50 | GS | 0.388845 | 0.21053 | 0.567164 |
| TaoWu | PPMI | gnb_pooled | 100 | GS | 0.512962 | 0.47368 | 0.552239 |
| TaoWu | PPMI | svm_linear | 100 | GS | 0.535546 | 0.52632 | 0.544776 |
| TaoWu | PPMI | gnb_pooled | 500 | GS | 0.630204 | 0.89474 | 0.365672 |
| TaoWu | PPMI | svm_linear | 500 | GS | 0.532011 | 0.57895 | 0.485075 |
| TaoWu | PPMI | gnb_pooled | 5000 | GS | 0.577965 | 0.89474 | 0.261194 |
| TaoWu | PPMI | svm_linear | 5000 | GS | 0.562451 | 0.73684 | 0.38806 |
| NEUROCON | PPMI | gnb_pooled | 10 | bandpass | 0.519049 | 0.10526 | 0.932836 |
| NEUROCON | PPMI | svm_linear | 10 | bandpass | 0.437156 | 0.1579 | 0.716418 |
| NEUROCON | PPMI | gnb_pooled | 50 | bandpass | 0.459348 | 0.10526 | 0.813433 |
| NEUROCON | PPMI | svm_linear | 50 | bandpass | 0.462883 | 0.05263 | 0.873134 |
| NEUROCON | PPMI | gnb_pooled | 100 | bandpass | 0.467203 | 0.21053 | 0.723881 |
| NEUROCON | PPMI | svm_linear | 100 | bandpass | 0.440888 | 0.1579 | 0.723881 |
| NEUROCON | PPMI | gnb_pooled | 500 | bandpass | 0.425766 | 0.10526 | 0.746269 |
| NEUROCON | PPMI | svm_linear | 500 | bandpass | 0.481932 | 0.1579 | 0.80597 |
| NEUROCON | PPMI | gnb_pooled | 5000 | bandpass | 0.414375 | 0.05263 | 0.776119 |
| NEUROCON | PPMI | svm_linear | 5000 | bandpass | 0.497054 | 0.21053 | 0.783582 |
| NEUROCON | TaoWu | gnb_pooled | 10 | bandpass | 0.475 | 0.15 | 0.8 |
| NEUROCON | TaoWu | svm_linear | 10 | bandpass | 0.525 | 0.35 | 0.7 |
| NEUROCON | TaoWu | gnb_pooled | 50 | bandpass | 0.475 | 0.35 | 0.6 |
| NEUROCON | TaoWu | svm_linear | 50 | bandpass | 0.5 | 0.25 | 0.75 |
| NEUROCON | TaoWu | gnb_pooled | 100 | bandpass | 0.525 | 0.45 | 0.6 |
| NEUROCON | TaoWu | svm_linear | 100 | bandpass | 0.575 | 0.35 | 0.8 |
| NEUROCON | TaoWu | gnb_pooled | 500 | bandpass | 0.475 | 0.35 | 0.6 |
| NEUROCON | TaoWu | svm_linear | 500 | bandpass | 0.7 | 0.4 | 1 |
| NEUROCON | TaoWu | gnb_pooled | 5000 | bandpass | 0.5 | 0.5 | 0.5 |
| NEUROCON | TaoWu | svm_linear | 5000 | bandpass | 0.65 | 0.35 | 0.95 |
| PPMI | NEUROCON | gnb_pooled | 10 | bandpass | 0.448626 | 0.19355 | 0.703704 |
| PPMI | NEUROCON | svm_linear | 10 | bandpass | 0.464456 | 0.35484 | 0.574074 |
| PPMI | NEUROCON | gnb_pooled | 50 | bandpass | 0.469235 | 0.29032 | 0.648148 |
| PPMI | NEUROCON | svm_linear | 50 | bandpass | 0.432796 | 0.03226 | 0.833333 |
| PPMI | NEUROCON | gnb_pooled | 100 | bandpass | 0.459976 | 0.29032 | 0.62963 |
| PPMI | NEUROCON | svm_linear | 100 | bandpass | 0.483572 | 0.09677 | 0.87037 |
| PPMI | NEUROCON | gnb_pooled | 500 | bandpass | 0.485364 | 0.32258 | 0.648148 |
| PPMI | NEUROCON | svm_linear | 500 | bandpass | 0.488351 | 0.03226 | 0.944444 |
| PPMI | NEUROCON | gnb_pooled | 5000 | bandpass | 0.476105 | 0.32258 | 0.62963 |
| PPMI | NEUROCON | svm_linear | 5000 | bandpass | 0.481481 | 0 | 0.962963 |
| PPMI | TaoWu | gnb_pooled | 10 | bandpass | 0.475 | 0.2 | 0.75 |
| PPMI | TaoWu | svm_linear | 10 | bandpass | 0.55 | 0.5 | 0.6 |
| PPMI | TaoWu | gnb_pooled | 50 | bandpass | 0.5 | 0.4 | 0.6 |
| PPMI | TaoWu | svm_linear | 50 | bandpass | 0.475 | 0.15 | 0.8 |
| PPMI | TaoWu | gnb_pooled | 100 | bandpass | 0.475 | 0.45 | 0.5 |
| PPMI | TaoWu | svm_linear | 100 | bandpass | 0.45 | 0.2 | 0.7 |
| PPMI | TaoWu | gnb_pooled | 500 | bandpass | 0.475 | 0.4 | 0.55 |
| PPMI | TaoWu | svm_linear | 500 | bandpass | 0.525 | 0.1 | 0.95 |
| PPMI | TaoWu | gnb_pooled | 5000 | bandpass | 0.45 | 0.45 | 0.45 |
| PPMI | TaoWu | svm_linear | 5000 | bandpass | 0.55 | 0.1 | 1 |
| TaoWu | NEUROCON | gnb_pooled | 10 | bandpass | 0.543309 | 0.29032 | 0.796296 |
| TaoWu | NEUROCON | svm_linear | 10 | bandpass | 0.515532 | 0.29032 | 0.740741 |
| TaoWu | NEUROCON | gnb_pooled | 50 | bandpass | 0.515532 | 0.29032 | 0.740741 |
| TaoWu | NEUROCON | svm_linear | 50 | bandpass | 0.364994 | 0.32258 | 0.407407 |
| TaoWu | NEUROCON | gnb_pooled | 100 | bandpass | 0.499403 | 0.25807 | 0.740741 |
| TaoWu | NEUROCON | svm_linear | 100 | bandpass | 0.41129 | 0.32258 | 0.5 |
| TaoWu | NEUROCON | gnb_pooled | 500 | bandpass | 0.506272 | 0.29032 | 0.722222 |
| TaoWu | NEUROCON | svm_linear | 500 | bandpass | 0.506272 | 0.29032 | 0.722222 |
| TaoWu | NEUROCON | gnb_pooled | 5000 | bandpass | 0.503883 | 0.32258 | 0.685185 |
| TaoWu | NEUROCON | svm_linear | 5000 | bandpass | 0.490143 | 0.25807 | 0.722222 |
| TaoWu | PPMI | gnb_pooled | 10 | bandpass | 0.568932 | 0.47368 | 0.664179 |
| TaoWu | PPMI | svm_linear | 10 | bandpass | 0.531422 | 0.42105 | 0.641791 |
| TaoWu | PPMI | gnb_pooled | 50 | bandpass | 0.607031 | 0.68421 | 0.529851 |
| TaoWu | PPMI | svm_linear | 50 | bandpass | 0.480754 | 0.84211 | 0.119403 |
| TaoWu | PPMI | gnb_pooled | 100 | bandpass | 0.621956 | 0.68421 | 0.559701 |
| TaoWu | PPMI | svm_linear | 100 | bandpass | 0.454635 | 0.84211 | 0.067164 |
| TaoWu | PPMI | gnb_pooled | 500 | bandpass | 0.652003 | 0.73684 | 0.567164 |
| TaoWu | PPMI | svm_linear | 500 | bandpass | 0.473095 | 0.78947 | 0.156716 |
| TaoWu | PPMI | gnb_pooled | 5000 | bandpass | 0.633346 | 0.73684 | 0.529851 |
| TaoWu | PPMI | svm_linear | 5000 | bandpass | 0.547722 | 0.78947 | 0.30597 |

## Consensus NMF clustering

For a more direct graphical depiction of the heterogeneity of the functional connectomes of the PD patient scans, we have applied *consensus NMF clustering* [Brunet et al., 2004] for a progressively increasing number of clusters *k*=2,…,18 (Fig C). The Figure depicts the symmetric consensus co-clustering matrices for the PD scans from the NEUROCON dataset. Note that besides the consistent grouping of the replicate scan pairs for each patient, it is difficult to single out an optimal number of clusters *k*.


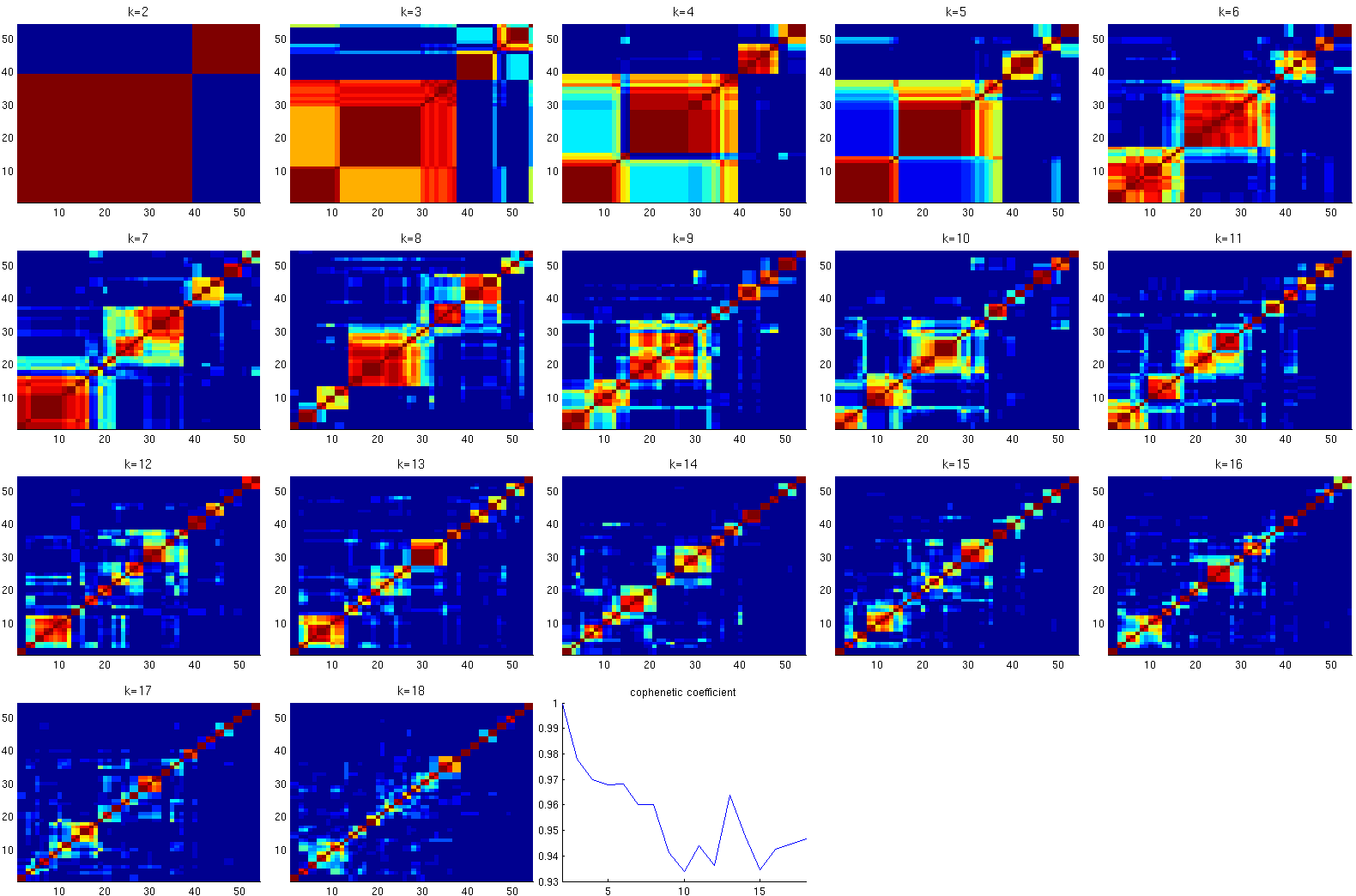


**Fig C. Consensus NMF clustering of functional connectomes of PD patient scans from the NEUROCON dataset.**

## Supplementary References

Brunet JP, Tamayo P, Golub TR, Mesirov JP. Metagenes and molecular pattern discovery using matrix factorization. Proceedings of the national academy of sciences. 2004 Mar 23;101(12):4164-9.

Fahn S. Recent developments in Parkinson's disease. Edited by S. Fahn, C. D. Mardsen, P. Jenner, and P. Teychenne. Raven Press; 1986.

Hoehn MM, Yahr MD. Parkinsonism: Onset, progression and mortality. Neurology 57:S11–S26. 2001.
